# Spatiotemporal clusters of Kawasaki disease in South Korea from 2008 to 2017: A municipal-level ecological study

**DOI:** 10.3389/fped.2022.1054985

**Published:** 2023-01-24

**Authors:** Jeehyun Kim, Kwan Hong, Daesung Yoo, Byung Chul Chun

**Affiliations:** ^1^ Department of Preventive Medicine, Korea University College of Medicine, Seoul, Republic of Korea; ^2^ Transdisciplinary Major in Learning Health Systems, Department of Healthcare Sciences, Graduate School, Korea University, Seoul, Republic of Korea; ^3^ Department of Animal Disease Control and Quarantine, Animal and Plant Quarantine Agency, Gimcheon, Republic of Korea

**Keywords:** Kawasaki disease, vasculitis, spatiotemporal analysis, cluster analysis, ecology

## Abstract

**Introduction:**

As the etiology of Kawasaki disease (KD) remains unknown, identifying spatiotemporal clusters with proper stratification of KD could provide further evidence for investigating the triggers of KD. However, spatiotemporal distributions of KD with sex stratification have never been reported. Therefore, we aimed to analyze the spatiotemporal patterns of KD by sex in South Korea.

**Methods:**

We extracted epidemiologic week (Epiweek)-based KD cases in patients <5 years of age (ICD-10-CM code: M303) from 2008 to 2017 national health insurance service data at the 250 municipal level. To determine whether spatial autocorrelation and persistent municipal-level clusters exist, year- and sex-stratified global Moran's *I* statistics, Getis-Ord Gi* statistics, and emerging hotspot analysis on KD incidence were conducted.

**Results:**

A total of 72,510 KD cases were reported between 2008 and 2017 (male-to-female ratio = 1.40:1). Incidence has increased since 2008, with the highest incidence in 2016 (396.8 per 100,000 population). KD had seasonality of winter and summer but different by sex. Positive spatial autocorrelation was consistently reported in every stratum, with the 2011–2014 period having the strongest index value (total sex *I *= 0.286, *p* < 0.001; male *I *= 0.242, *p* < 0.001; female *I *= 0.213, *p* < 0.001). Hot spots were consistently detected in the northern parts, and cold spots were in the southern part for 9 years in both sexes. The emerging hot spot analysis showed new, consecutive, and sporadic hot spots on the northwestern and eastern coasts and new and sporadic cold spots in the southwestern part. However, the distribution and proportion of hot or cold spot types differed according to sex.

**Discussion:**

The spatiotemporal features of KD had limits to concluding that only infectious triggers result in KD occurrence. Therefore, our findings support the notion that KD is a syndrome with multiple factors, including infectious, genetic, and environmental factors, that are associated with sex differences.

## 1. Introduction

Kawasaki disease (KD) is an idiopathic systemic vasculitis that initially manifests in the form of high fever and mucocutaneous inflammation, particularly affecting infants and young children (1). While the disease has been a leading cause of cardiac disease in children in developed countries (1, 2), an increasing incidence of KD has been reported in developing countries, including China and India (1). South Korea has the second-highest incidence of KD, following Japan (1). Although prompt diagnosis and treatment of KD are essential to lower the possibility of coronary artery abnormalities (2), the undefined etiology and pathogenesis hinder the exact diagnosis and prevention of KD (1, 2).

Although numerous reports have been published, the etiology of KD remains elusive. The prominent paradigm is that an immunological reaction is caused in genetically susceptible hosts upon exposure to infectious agents (3, 4). However, multifactorial etiology, which includes environmental factors in addition to genetic and environmental factors, has also been suggested (5, 6). Studies using spatial and temporal cluster analyses of KD could provide further evidence to investigate the KD etiology (7). In particular, spatiotemporal analysis is required for an accurate description of disease distribution and transmission patterns (8), as the results could lead to valuable insights into disease causality. However, most previous studies have limitations in settling on a temporal perspective without considering a spatial perspective (9–13) and some studies with spatiotemporal axes have limitations in exploring national representative data (14–17). Furthermore, studies with sex stratification could provide evidence for the etiology. However, as Burns criticized, the urge to detect a single trigger in a whole population without stratification could have led to KD etiology being undiscovered. Therefore, our study aimed to analyze the spatiotemporal patterns of KD with sex stratification in South Korea using nationally representative data.

## 2. Materials and methods

### 2.1. Study design and participants

We extracted epidemiologic week (Epiweek)-based KD cases from 2008 to 2017 National Health Insurance Service (NHIS) data at the municipal level and excluded cases in patients who were not under 5 years of age, did not have a municipal code, or were not included in the study period. KD was defined according to the International Classification of Diseases, Tenth Revision, Clinical Modification code (ICD-10-CM code) of M303.

We set the year-time period based on the seasonality of KD (2). For example, the period from 2008 to 2009 starts at 2008 Epiweek 12 and ends at 2009 Epiweek 11. Because there were no data in 2018, year-time period 2017 ended at 2017 Epiweek 52. Considering the trend of KD that increases every 3 years, spatial autocorrelation and spatial cluster were analyzed with 3-year accumulated KD incidence (e.g., 2008 Epiweek 12 to 2011 Epiweek 11). However, for comparison with previous studies, a descriptive analysis was performed with KD cases from 2008 to 2017 in the conservative calendar year (e.g., 2008 Epiweek 1 to 2008 Epiweek 53). We analyzed the KD incidence per 100,000 people using population data from Statistics Korea (https://kosis.kr).

We used 250 municipal-level administrative boundaries in 2017 as the standard analysis unit, and the incidence in 2008–2016 was modified to fit the analysis unit. For the spatial analysis, we obtained a municipal-level shapefile from the Statistical Geographic Information Service (https://sgis.kostat.go.kr), which is publicly accessible. The minimum distance between the closest municipalities was 1,541.2 m, and the average distance was 15,031.5 m. The Institutional Review Board (IRB) of Korea University granted an exemption for this study (IRB exemption number: KUIRB-2021-0237-02) because the data did not contain any personal identification information.

### 2.2. Statistical analysis

#### 2.2.1. Descriptive analysis

Case count and KD incidence per 100,000 population were determined in terms of sex and years of age by conservative calendar year to provide the basic information and explore sex and age difference by the calendar year in KD. Weekly temporal graphs and their decomposition on incidence per 100,000 population were recorded using an additive model in sex stratification to determine and compare the trend and seasonality by sex using R version 4.0.3 (R Foundation, Vienna, Austria, https://cran.r-project.org/).

#### 2.2.2. Spatial analysis

##### 2.2.2.1. Choropleth maps and global Moran's ***I*** **statistics**

Choropleth maps of the incidence with ten geometric intervals were depicted under a year and sex stratification to determine the spatiotemporal distribution of KD incidence. In addition, the yearly male-to-female ratio of KD incidence in each municipality was depicted to detect the sex difference of KD cases along the spatial axis. The municipality with a value above 1 implies that the district had a higher KD incidence of males than females, and the higher the value, the greater the difference in incidence between males and females. On the other hand, the district with a value less than 1 indicates a higher incidence of females than males, and the lower the value, the greater the difference in incidence between males and females. Municipalities without female cases were given an “out-of-range” value.

To determine whether spatial autocorrelation exists, we performed global Moran's *I* statistics (18) on 3-year accumulated KD incidence and sex stratification with row-standardized spatial weights. The municipalities inside the critical distance in the Euclidean distance are defined as neighbors, and these municipalities obtain a spatial weight of 1, while others obtain a spatial weight of zero. The cutoff distance was the average value—15,031.484037 m—calculated from the “Calculate Distance Band from Neighbor Count” tool. When calculating the cutoff value, the neighbor parameter was specified as 1, which means that every centroid of the polygon would find its first nearest-neighbor on average with this cutoff distance. The global Moran's *I* statistics (I) is given by the following formula:

$$I=\frac{n}{S_{0}}\frac{\sum_{i=1}^{n}\sum_{\;j=1}^{n}w_{i,j}z_{i}z_{j}}{\sum_{i=1}^{n}z_{i}^{2}}\;\;\;S_{0}=\overset{n}{\underset{i=1}{\sum }}\overset{n}{\underset{\;j=1}{\sum }}w_{i,j}$$

where *n* is equal to the total number of municipalities, $z_{i}$ is the deviation of an attribute for future *i* from its mean, $w_{i,j}$ is the spatial weight between municipalities *i* and *j*, and $S_{0}$ is an aggregate of all spatial weights.

The results of Moran's *I* statistics have an index value *I*, and the difference between the expected and observed values of *I* is evaluated with a 95% confidence level. The index value usually ranges from −1 to 1. A positive value implies the presence of positive autocorrelation, meaning that the attributes are spatially clustered. A zero value implies the absence of spatial autocorrelation. When the index value is statistically significant, the null hypothesis, meaning that the attribute has complete spatial randomness, can be rejected.

##### 2.2.2.2. Getis-Ord Gi* analysis

We performed a Getis-Ord Gi* analysis (18) with year and sex stratification to determine whether municipal-level clusters differ yearly and by sex. The Getis-Ord local statistics $\left(G_{i}^{*}\right)$, itself being the *z*-score, is given as follows:

$$G_{i}^{*}=\frac{\sum_{\;j=1}^{n}w_{i,j}x_{j}-\bar{X}\sum_{\;j=1}^{n}w_{i,j}}{S\sqrt{\frac{\left[n\sum_{\;j=1}^{n}w_{i,j}^{2}-{\left(\sum_{\;j=1}^{n}w_{i,j}\right)}^{2}\right]}{n-1}}}$$

where *n* is the total number of municipalities, $w_{i,j}$ is the spatial weight between municipalities *i* and *j*, and $x_{j}$ is the attribute value for municipality *j*. $\bar{X}$ and *S* are defined as follows:

$$\bar{X}=\frac{\sum_{\;j=1}^{n}x_{j}}{n},S=\sqrt{\frac{\sum_{\;j=1}^{n}x_{j}^{2}}{n}-{\left(\bar{X}\right)}^{2}}$$

Municipality with a high positive and statistically significant $G_{i}^{*}$ statistics value indicates spatial clustering of high values, i.e., “hot spots.” A municipality that is a hot spot has a high value and is surrounded by other municipalities with high values. In contrast, a “cold spot” has a low negative and statistically significant $G_{i}^{*}$ statistics value, that is, spatial clustering of low values. A cold spot municipality has a low value and is surrounded by other municipalities with low values. The results are given with three confidence levels for hot or cold spots: 90%, 95%, and 99%.

##### 2.2.2.3. Emerging hot spot analysis

Emerging hot spot analysis (EHSA) (19) was performed under sex stratification to determine whether statistically significant spatiotemporal patterns existed (20, 21). EHSA is a combination of two statistical measures: (1) the Getis-Ord Gi* statistics to identify the location and intensity of the spatial cluster of KD incidence and (2) the Mann–Kendall statistics to evaluate temporal trends across the time series.

Before conducting EHSA, a space–time cube in the network Common Data Form (netCDF) was created with 250 municipal polygons as a spatial unit and 1 year-time period as a temporal unit using the “Create Space Time Cube From Defined Locations” tool. Thus, the space–time cube was a three-dimensional cube (x,y,t), in which the *x* and *y* dimensions, representing the space, were stacked in time axis *t* (250 spatial units × 10 temporal units = 2,500 bins). Each bin holds attribute information (i.e., KD incidence) from each municipality for each year. The missing values of the attributes of each bin were replaced with zero.

For the first step of EHSA, each bin was given a *z*-score resulting from the Gets-Ord Gi* statistics. Second, the temporal trends of the *z*-score of each bin were evaluated by comparing the score of each time step to the score of the one-time step after it. Finally, each pair of time steps in each bin was compared to the 10-year time series using the Mann–Kendall statistics, which resulted in trend *z*-scores and *p*-values for each bin. Based on the variance of the values in the bin time series and the number of time units, the observed sum was compared to the expected sum to determine whether the difference was statistically significant with a 95% confidence level. The expected sum was zero, indicating no trend in the values over time.

The results were then categorized into 17 patterns, including eight patterns of hot spots (new, consecutive, intensifying, persistent, diminishing, sporadic, oscillating, and historical), eight patterns of cold spots (new, consecutive, intensifying, persistent, diminishing, sporadic, oscillating, and historical), and one without any pattern (Supplementary Table S1). For example, a municipality with a new hot (or cold) spot has a statistically significant hot (or cold) spot at the final time step without having a statistically significant hot (or cold) spot before the final time step. Consecutive hot (or cold) spots refer to municipalities with a single uninterrupted run of at least two statistically significant hot (or cold) spots in the final time step without a statistical significance before. Sporadic hot (or cold) spots indicated that corresponding municipalities were detected as hot (or cold) spots on and off again.

For sensitivity analysis, Getis-Ord Gi* cluster analysis was conducted by year. In addition, EHSA was performed (1) weekly from 2008 Epiweek 12 to 2017 Epiweek 52 (t=511) and (2) weekly from 2008 Epiweek 12 to 2017 Epiweek 11 (t=470) because we lack the data for 2018 to make the 2017–2018 year-time period complete. In addition, EHSA was performed using a row-standardized spatial weight matrix with the K nearest-neighbor set to 2 to ensure every municipality has two neighborhoods. All spatial analyses, including mapping, determining spatial autocorrelation, and clusters, were performed using ArcGIS Pro version 2.9.2 (Esri, West Redlands, CA, United States, https://www.esri.com).

## 3. Results

### 3.1. Descriptive analysis

**Figure 1:**
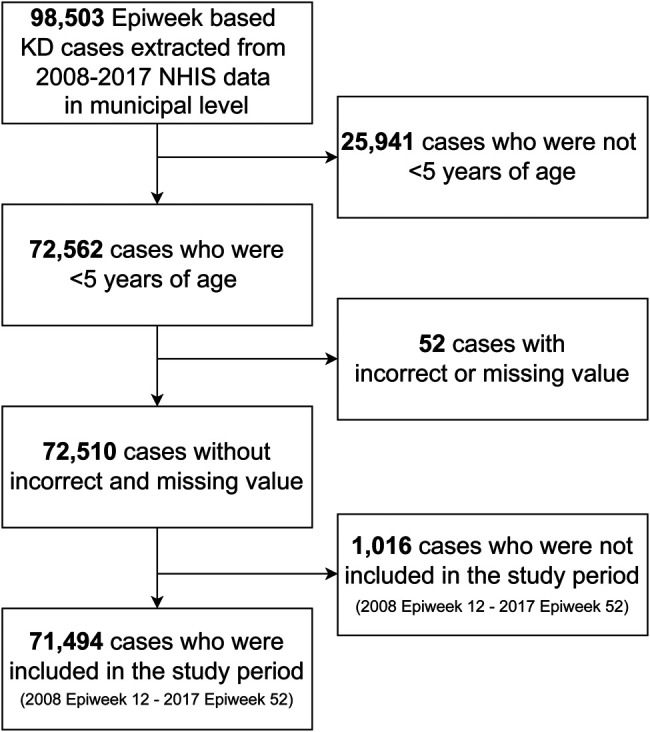
Flowchart of study subject selection. Kawasaki disease was diagnosed with M303 in ICD-10-Code. Epiweek, Epidemiologic week. KD, Kawasaki disease. NHIS, National Health Insurance Service in South Korea.

We extracted 98,503 Epiweek-based KD cases from 2008 to 2017 NHIS data at the municipal level. A total of 72,510 patients who were <5 years of age and without incorrect or missing values were included in descriptive analysis, and 71,494 patients with only cases within the study period were included in the spatial analysis (Figure 1).

Table 1 presents the case count and incidence of KD by conservative calendar year. In the total study subjects, the incidence per 100,000 population aged <5 years has increased by approximately 147.2% from 2008 to 2017 (374.5 in 2017; 254.5 in 2008). The incidence of KD highly increased in females (160.0%; 209.2 in 2008; 334.7 in 2017) than in males (138.9%; 297.0 in 2008; 412.4 in 2017). The highest incidence was in 2016 in total subjects and both sexes (total sex, 396.8; male, 453.1; female, 337.5). The incidence of KD in males was consistently higher than in females from 2008 to 2017.

**Table 1:** Case count and incidence per 100,000 population of Kawasaki disease by calendar year.

	Case count, *N* (%)				Incidence per 100 000 population			
Year	Total sex	Male	Female	Male-to-female ratio	Total sex	Male	Female	Male-to-female ratio
2008	5,829 (100.0)	3,514 (60.3)	2,315 (39.7)	1.52	254.5	297.0	209.2	1.42
2009	6,039 (100.0)	3,540 (58.6)	2,499 (41.4)	1.42	266.8	303.2	228.1	1.33
2010	7,227 (100.0)	4,255 (58.9)	2,972 (41.1)	1.43	314.3	358.9	266.7	1.35
2011	6,855 (100.0)	3,982 (58.1)	2,873 (41.9)	1.39	294.4	332.2	254.3	1.31
2012	6,977 (100.0)	4,056 (58.1)	2,921 (41.9)	1.39	300.6	339.6	259.2	1.31
2013	8,097 (100.0)	4,787 (59.1)	3,310 (40.9)	1.45	351.8	404.6	296.0	1.37
2014	7,511 (100.0)	4,361 (58.1)	3,150 (41.9)	1.38	327.4	370.1	282.3	1.31
2015	7,442 (100.0)	4,253 (57.1)	3,189 (42.9)	1.33	328.3	365.8	288.9	1.27
2016	8,746 (100.0)	5,120 (58.5)	3,626 (41.5)	1.41	396.8	453.1	337.5	1.34
2017	7,787 (100.0)	4,397 (56.5)	3,390 (43.5)	1.30	374.5	412.4	334.7	1.23
Total	72,510 (100.0)	42,265 (58.3)	30,245 (41.7)	1.40				

The male-to-female ratio of the total case count was 1.40:1 [males, 42,265 (58.3%); females, 30,245 (41.7%)]. However, the male-to-female ratios of case count and incidence declined from 2008 to 2017. The mean patient age was 2.0 for males and 2.1 for females. The proportion of 1-year olds was the highest among the study subjects and both sexes [total sex: 20,846 (28.7%); males: 12,414 (29.4%); females: 8,432 (27.9%)], followed by the proportion of 2-year olds [total sex: 17,442 (24.1%); males: 10,119 (23.9%); females: 7,323 (24.2%), Table 2]. The male-to-female ratio of the case count was the highest in 0-year olds (1.57) and decreased as age increased (1 year olds, 1.47; 2 year olds, 1.38; 3 year olds, 1.31; 4 year olds, 1.32).

**Table 2:** Case count of Kawasaki disease by sex, years of age, and calendar year, and the male-to-female ratio by calendar year.

	Total sex						Male						Female					
	Years of age, *N* (%)						Years of age, *N* (%)						Years of age, *N* (%)					
Year	0	1	2	3	4	Total	0	1	2	3	4	Total	0	1	2	3	4	Total
2008	613 (10.5)	1,843 (31.6)	1,269 (21.8)	1,104 (18.9)	1,000 (17.2)	5,829 (100.0)	390 (11.1)	1,127 (32.1)	771 (21.9)	614 (17.5)	612 (17.4)	3,514 (100.0)	223 (9.6)	716 (30.9)	498 (21.5)	490 (21.2)	388 (16.8)	2,315 (100.0)
2009	541 (9.0)	1,814 (30.0)	1,511 (25.0)	1,189 (19.7)	984 (16.3)	6,039 (100.0)	326 (9.2)	1,072 (30.3)	890 (25.1)	679 (19.2)	573 (16.2)	3,540 (100.0)	215 (8.6)	742 (29.7)	621 (24.8)	510 (20.4)	411 (16.4)	2,499 (100.0)
2010	632 (8.7)	1,926 (26.7)	1,744 (24.1)	1,661 (23.0)	1,264 (17.5)	7,227 (100.0)	390 (9.2)	1,153 (27.1)	1,024 (24.1)	940 (22.1)	748 (17.6)	4,255 (100.0)	242 (8.1)	773 (26.0)	720 (24.2)	721 (24.3)	516 (17.4)	2,972 (100.0)
2011	671 (9.8)	2,096 (30.6)	1,586 (23.1)	1,332 (19.4)	1,170 (17.1)	6,855 (100.0)	410 (10.3)	1,241 (31.2)	927 (23.3)	746 (18.7)	658 (16.5)	3,982 (100.0)	261 (9.1)	855 (29.8)	659 (22.9)	586 (20.4)	512 (17.8)	2,873 (100.0)
2012	713 (10.2)	2,031 (29.1)	1,665 (23.9)	1,348 (19.3)	1,220 (17.5)	6,977 (100.0)	414 (10.2)	1,208 (29.8)	932 (23.0)	787 (19.4)	715 (17.6)	4,056 (100.0)	299 (10.2)	823 (28.2)	733 (25.1)	561 (19.2)	505 (17.3)	2,921 (100.0)
2013	660 (8.2)	2,260 (27.9)	1,956 (24.2)	1,726 (21.3)	1,495 (18.5)	8,097 (100.0)	392 (8.2)	1,338 (28.0)	1,180 (24.7)	1,018 (21.3)	859 (17.9)	4,787 (100.0)	268 (8.1)	922 (27.9)	776 (23.4)	708 (21.4)	636 (19.2)	3,310 (100.0)
2014	681 (9.1)	2,058 (27.4)	1,926 (25.6)	1,598 (21.3)	1,248 (16.6)	7,511 (100.0)	436 (10.0)	1,234 (28.3)	1,120 (25.7)	881 (20.2)	690 (15.8)	4,361 (100.0)	245 (7.8)	824 (26.2)	806 (25.6)	717 (22.8)	558 (17.7)	3,150 (100.0)
2015	729 (9.8)	2,056 (27.6)	1,756 (23.6)	1,610 (21.6)	1,291 (17.3)	7,442 (100.0)	422 (9.9)	1,248 (29.3)	976 (22.9)	924 (21.7)	683 (16.1)	4,253 (100.0)	307 (9.6)	808 (25.3)	780 (24.5)	686 (21.5)	608 (19.1)	3,189 (100.0)
2016	785 (9.0)	2,411 (27.6)	2,073 (23.7)	1,789 (20.5)	1,688 (19.3)	8,746 (100.0)	508 (9.9)	1,422 (27.8)	1,209 (23.6)	1,026 (20.0)	955 (18.7)	5,120 (100.0)	277 (7.6)	989 (27.3)	864 (23.8)	763 (21.0)	733 (20.2)	3,626 (100.0)
2017	709 (9.1)	2,351 (30.2)	1,956 (25.1)	1,500 (19.3)	1,271 (16.3)	7,787 (100.0)	424 (9.6)	1,371 (31.2)	1,090 (24.8)	811 (18.4)	701 (15.9)	4,397 (100.0)	285 (8.4)	980 (28.9)	866 (25.5)	689 (20.3)	570 (16.8)	3,390 (100.0)
Total	6,734 (9.3)	20,846 (28.7)	17,442 (24.1)	14,857 (20.5)	12,631 (17.4)	72,510 (100.0)	4,112 (9.7)	12,414 (29.4)	10,119 (23.9)	8,426 (19.9)	7,194 (17.0)	42,265 (100.0)	2,622 (8.7)	8,432 (27.9)	7,323 (24.2)	6,431 (21.3)	5,437 (18.0)	30,245 (100.0)
Male-to-female ratio	1.57	1.47	1.38	1.31	1.32	1.40												

**Figure 2:**
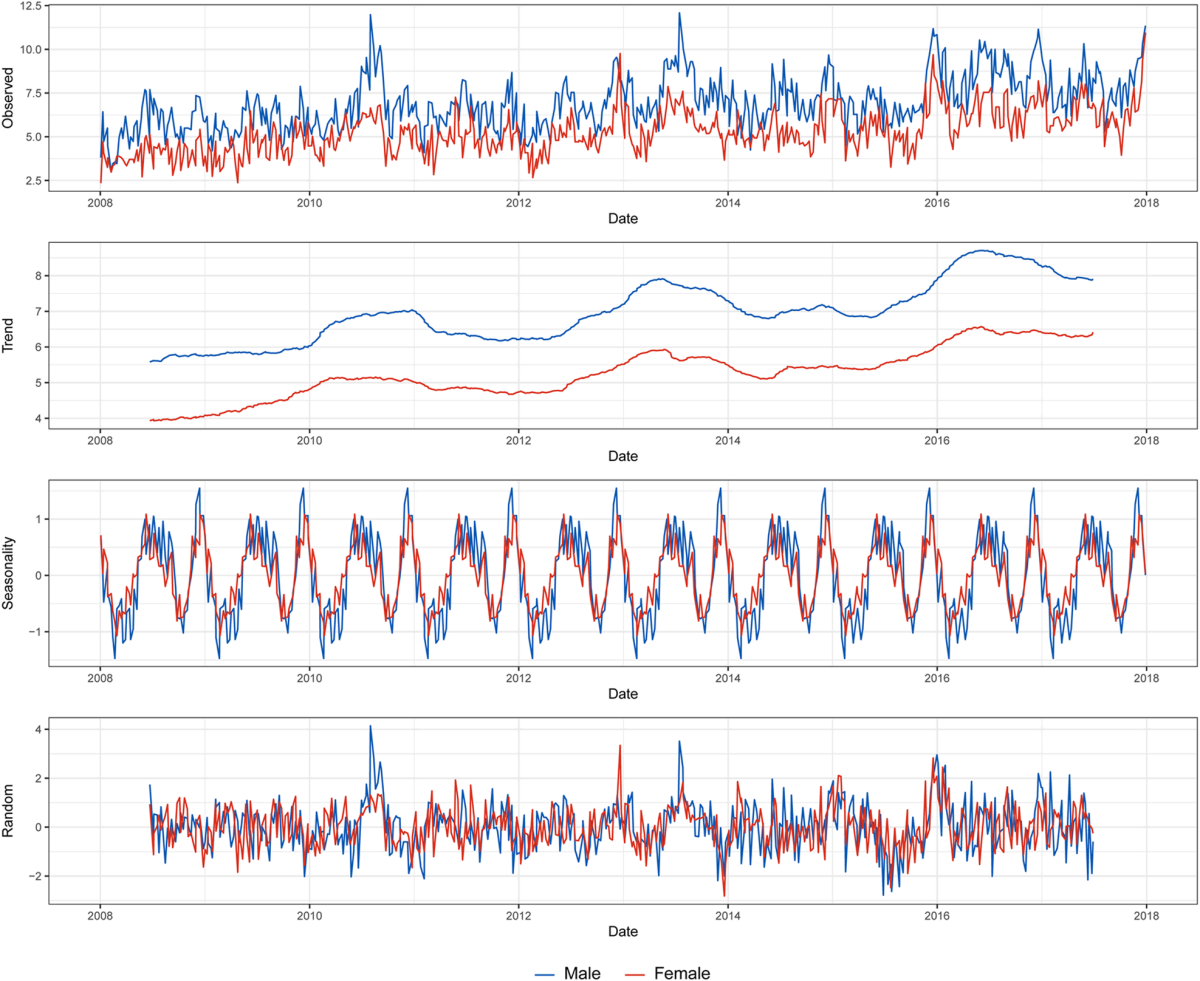
Decomposition of the weekly based Kawasaki disease incidence per 100 000 population by sex.

Figure 2 depicts the time series of the incidence by sex, including its trend, seasonality, and white noise. In both sexes, the incidence increased approximately every 3 years, and the seasonality in winter and summer was observed. While the peak of winter was higher than that of summer in males, the peaks of summer and winter were similar in females.

### 3.2. Spatial analysis

#### 3.2.1. Spatial descriptive analysis and spatial autocorrelation

**Figure 3:**
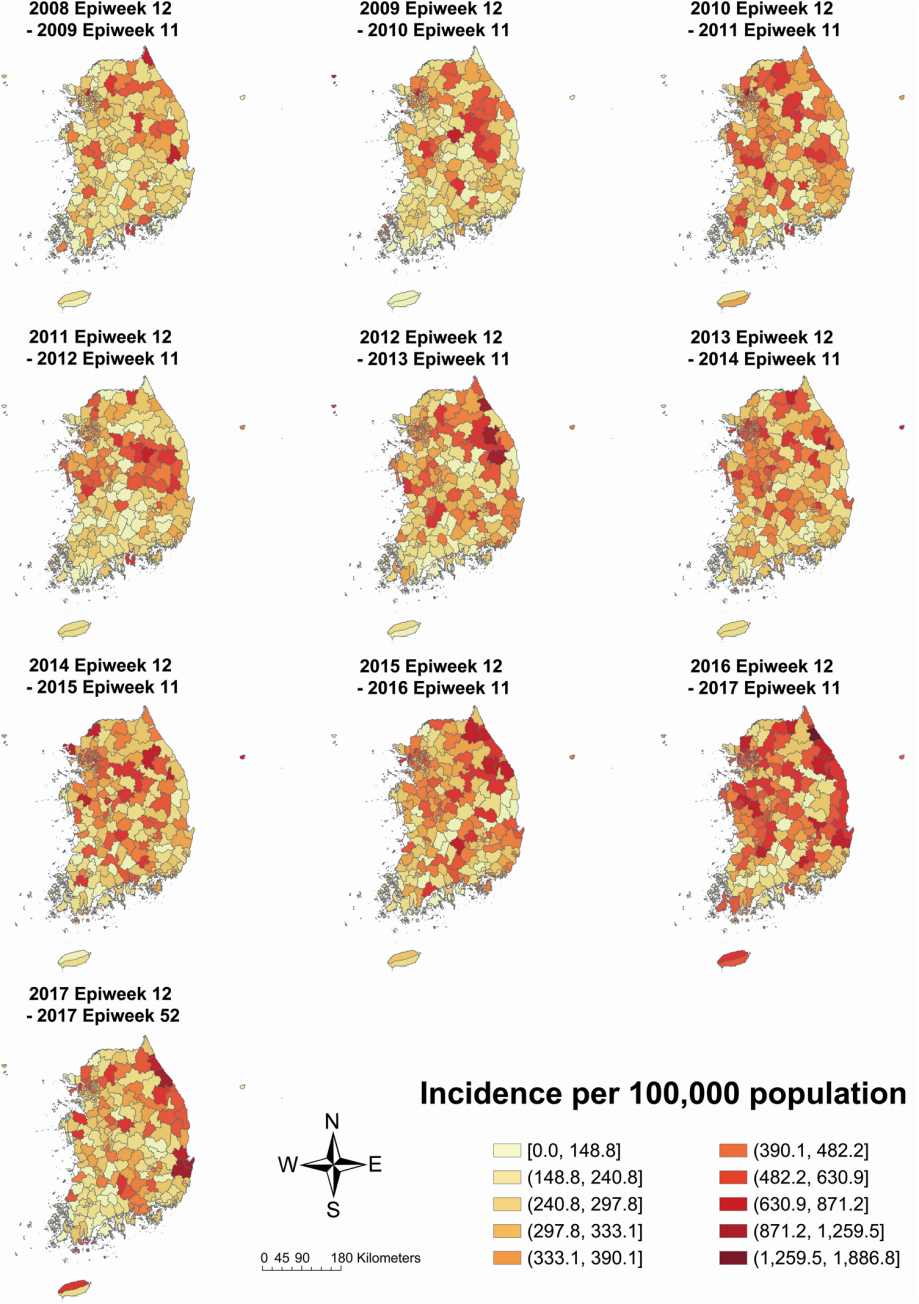
Kawasaki disease incidence per 100,000 population on total population by each year-time period. *The 2017 Epiweek 12 to 2017 Epiweek 52 period includes a partial year, i.e., 41 weeks*.

According to the incidence map by study period, municipalities with a high incidence were scattered across the middle and northern parts of South Korea (Figure 3). Specifically, the gathering of high incidence moved from the middle part (2011–2012 period) to the northeastern part (2012–2013 period) and the east coast (2016–2017 and 2017 periods). The incidence of Jeju-si and Seogwipo-si, which are municipalities of Jeju-do—the island located in the southern end of the Korean Peninsula, increased from 2008–2009 to 2016–2017 (Jeju-si, 156.4–587.2; Seogwipo-si, 121.6–462.1).

Compared to the incidence map of females, males had a more distinct gathering of high incidence in the mid-eastern part in the 2011–2012 period, the northeastern part in the 2012–2013 period, and the east coast in the 2016–2017 period but less distinct in the east coast and Jeju-do in the 2017 period (male: Supplementary Figure S1; female: Supplementary Figure S2). However, the incidence in Jeju-si and Seogwipo-si increased from 2008–2009 to 2016–2017 in both sexes (male Jeju-si 211.9–612.9; male Seogwipo-si 128.2–453.5; female Jeju-si 96.1–560.0; female Seogwipo-si 114.2–471.0).

**Figure 4:**
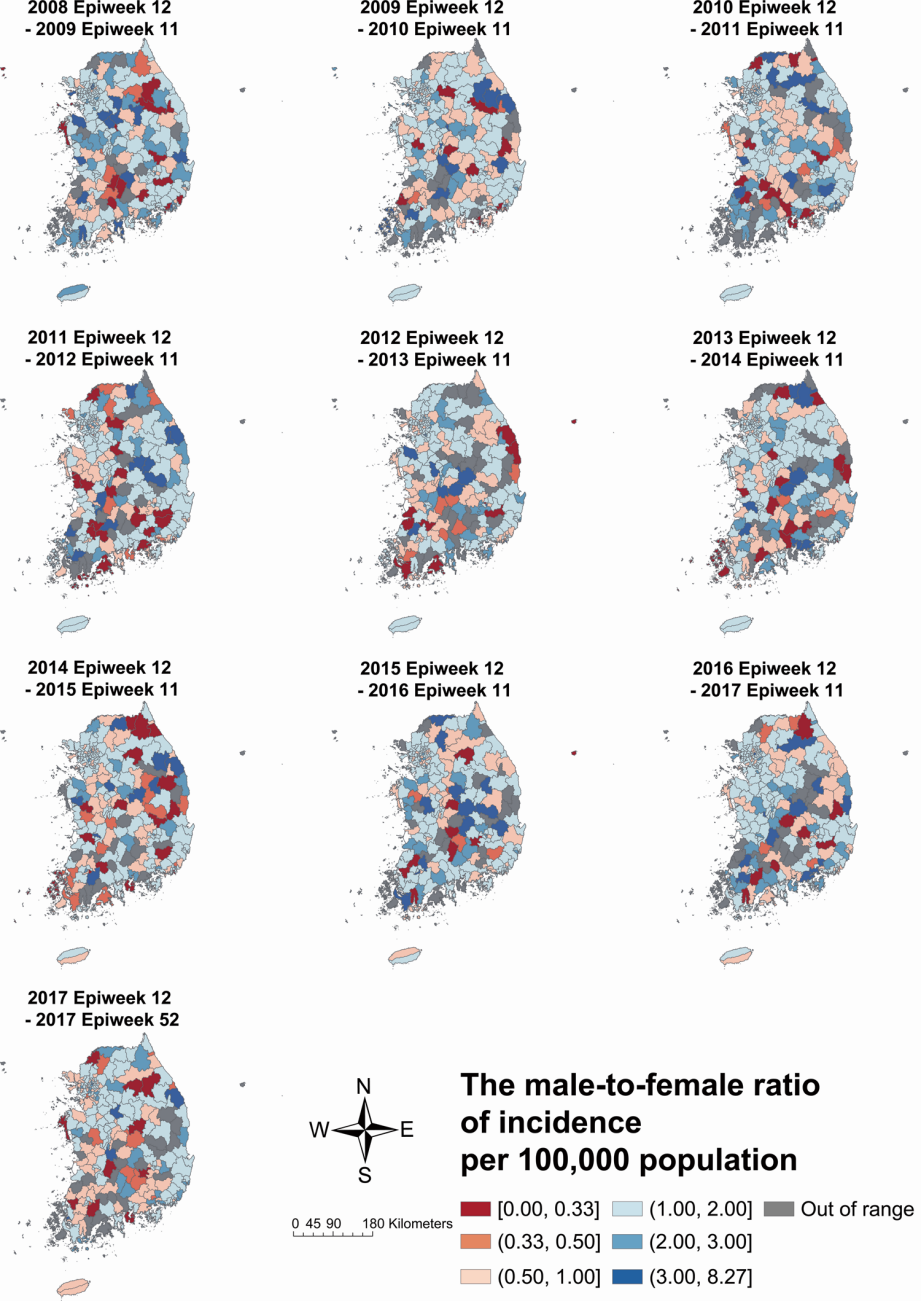
The male-to-female ratio of Kawasaki disease incidence per 100 000 population by each year-time period. *The 2017 Epiweek 12 to 2017 Epiweek 52 period includes a partial year, i.e., 41 weeks*.

The male-to-female ratios of KD incidence in each municipality by study period are depicted in Figure 4. Municipalities with higher or lower sex ratios scattered across the country from 2008–2009 to 2010–2011. From the 2011–2012 to 2014–2015 period and 2017 period, however, municipalities with higher sex ratios, which means male-dominant, were usually located in the mid-eastern part and female-dominant municipalities in the southwestern part. The incidence in Jeju-do was male-dominant from 2008–2009 to 2013–2014 period, but the sex ratio began to decline from the 2014–2015 period and eventually became female-dominant in the 2017 period.

Positive spatial autocorrelation was consistently reported in every stratum, with the 2011–2014 period having the strongest index value in total study subjects (*I *= 0.286, *p* < 0.001) and males (*I *= 0.242, *p* < 0.001), while the strongest in 2008–2011 period in females (*I *= 0.213, *p* < 0.001, Table 3).

**Table 3:** Results of global Moran's *I* statistics of Kawasaki disease by each time period.

Global Moran's index (*p*-value)			
Year	Total sex	Male	Female
2008–2011	**0.232** (**<0.001)**	**0.174** (**0.003)**	**0.213** (**<0.001)**
2011–2014	**0.286** (**<0.001)**	**0.242** (**<0.001)**	**0.151** (**0.010)**
2014–2017	**0.250** (**<0.001)**	**0.199** (**0.001)**	**0.147** (**0.013)**

*Values in bold indicate statistical significance.*

#### 3.2.2. Cluster analysis

**Figure 5:**
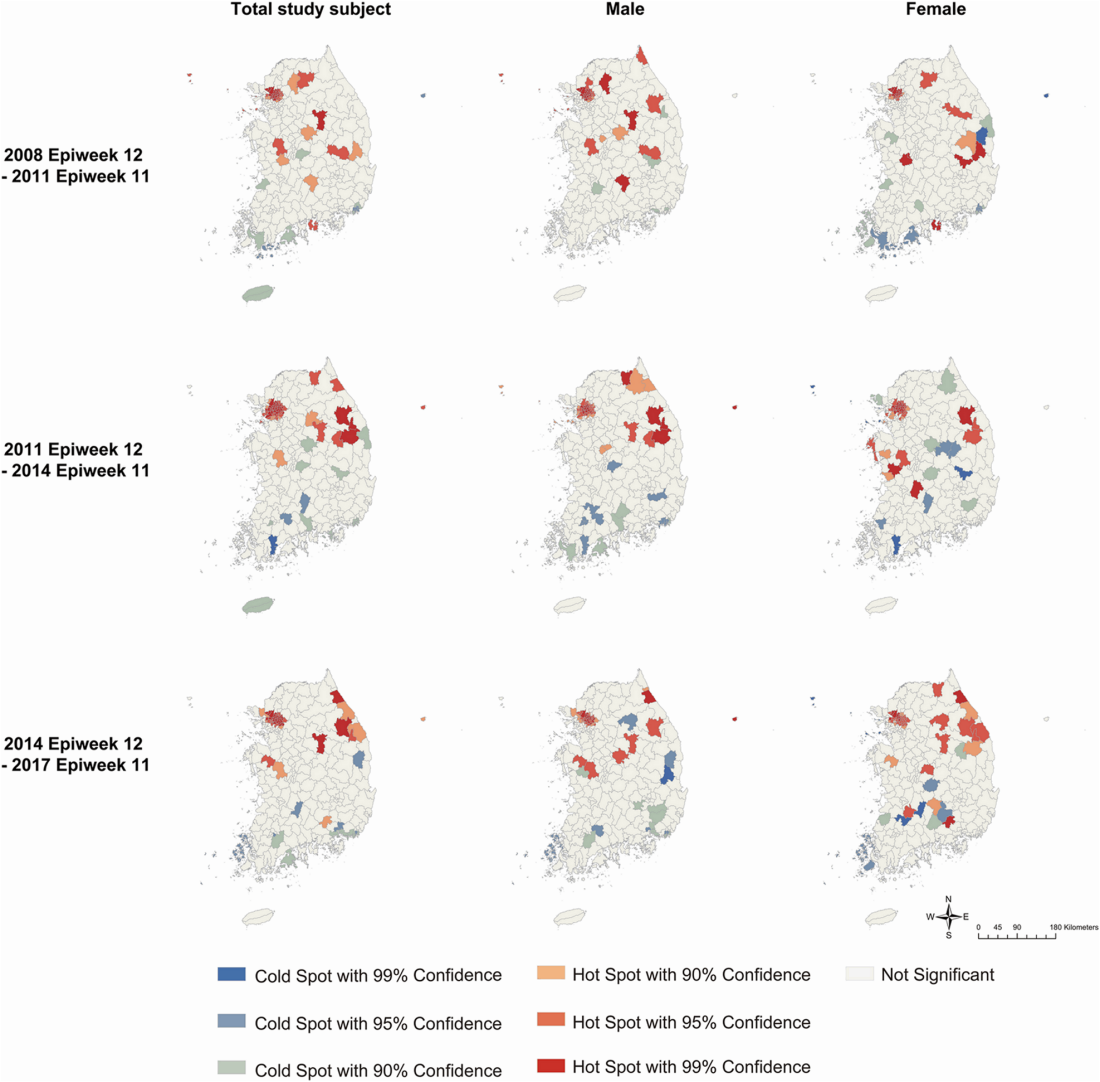
Clusters identified by Getis-Ord Gi* analysis for Kawasaki disease incidence per 100 000 population every three years by sex. *Colors represent hot spots and cold spots of spatial clustering with 90%, 95%, and 99% confidence intervals*.

According to the results of Getis-Ord Gi* analysis, hot spots were consistently detected in the northern part, including the capital area, for 9 years in both sexes (Figure 5). Cold spots were constantly observed in the southern part of both sexes. However, the specific municipalities with hot or cold spots differed every 3 years. In the total study subject, the gathering of hot spots moved from the middle part (2009–2011 period) to the northeastern part (2011–2014 period) and the east coast (2014–2017 period).

#### 3.2.3. Emerging hot spot analysis

**Figure 6:**
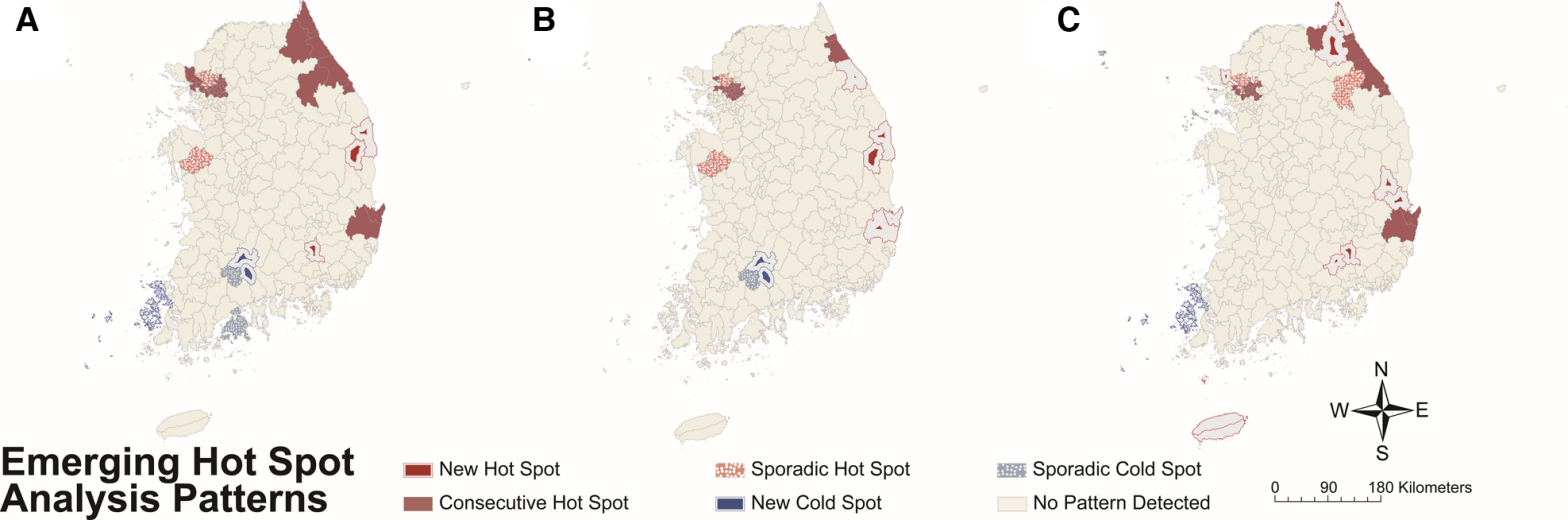
Hot or cold spot area detected by emerging hot spot analysis for Kawasaki disease incidence per 100,000 population by each year-time period on (**A**) total study subjects, (**B**) males, and (**C**) females. Colors represent the patterns of hot or cold spots (refer to Supplementary Table S1).

The results of EHSA by year-time period for total sex, males, and females are presented in Figure 6 and Supplementary Table S2. In total sex, new, consecutive, and sporadic hot spots were detected in the northwestern area, which is the capital area, and the east coast, and new and sporadic cold spots were detected in the southwestern part (Figure 6A). There were three new hot spots, 35 consecutive hot spots, 13 sporadic hot spots, 3 new cold spots, and 2 sporadic cold spots.

Although hot spots were detected on the east coast and northwestern part and cold spots in the southwestern part in both sexes, the distribution and proportion of the two types of spots differed. Males had a total of 37 hot spots, while females had a total of 45 hot spots. Unlike males, females had new hot spots in southeastern inland and Jeju-do. In addition, females had a higher proportion of new [male, 5 (13.5%); female, 9 (20.0%)] and sporadic [male, 7 (18.9%); female, 9 (20.0%)] hot spots among total hot spots compared to males (see Figures 6B, C for male and female data, respectively).

## 4. Discussion

This study is the first to analyze spatiotemporal patterns of KD incidence with sex stratification. We investigated the demographic, temporal, and spatial distributions, especially the statistically significant spatiotemporal clusters of KD incidence and differences between the sexes using national representative data.

The incidence of KD has increased steadily since 2008. This increasing trend in KD is consistent with previous studies (22, 23). Although the reason for the steady increase is unknown, it could result from a true increase in incidence or from physicians becoming more vigilant about the disease (23). Although we detected the highest incidence in 2016 (or the 2016–2017 period in the sensitivity analysis), a previous study reported that 2015 had the highest incidence. This difference could result from different characteristics that each database has; in particular, our data were extracted from the National Health Insurance Service data of Korea, where all healthcare providers and citizens should enroll mandatorily, while the previous study was based on data from some hospitals that participated in a certain survey (23). In addition, KD incidence had certain epidemiologic features compatible with an infectious etiology, such as 3-year interval increment and seasonal variation, following previous studies (4).

Municipal-level spatial autocorrelation was detected from 2008 to 2017. Therefore, KD outbreaks should be explored by considering spatial autocorrelation. Municipalities with high incidences and hot spots were found throughout the central and northern parts of South Korea. However, hot spots were consistently detected around the northwestern part, which is the capital area. Spatial scanning analysis results in Japan for 2007–2012 also detected the most likely clusters in the metropolitan area, which suggested that the area with a large population of children could be the reason for clusters (14), supporting infectious triggers. However, our study and that of Sano et al. also found hot spots in other municipalities, suggesting that factors other than the number of children in a population could also result in KD outbreaks.

Temporal changes in spatial patterns in KD could partly support the infectious trigger hypothesis. The EHSA detected new, consecutive, and sporadic hot spots without detecting intensifying or persistent hot spots, even when using diverse spatial weights or time criteria (Supplementary Figures S5–S7). Considering the results of EHSA on 4-month-accumulated COVID-19 cases, which is a typical infectious disease and also presents new, consecutive, and sporadic hot spots (24), it may be concluded that KD has characteristics of infectious diseases. However, the geographic wave-like spread was not detected in our study. The particular spread pattern was reported in Japan during the nationwide KD epidemic in 1985–1986 (25) and has been identified as a distinct epidemiologic feature of KD to support infectious trigger etiology (4). Although the difference could be derived from whether the spatial distribution was explored in an epidemic situation or not, there is a limit to concluding that KD results from a single infectious trigger. Furthermore, even in different time criteria, hot spots were consistently detected in the northeastern part and cold spots in the southwestern part (Supplementary Figures S3–S4). This could infer environmental factors, such as tropospheric winds (26), temperature (17, 27), and precipitation (27), or genetic factors should be suspected as possible factors.

Sex differences detected in KD also support the possibility of factors other than infectious triggers. The male-to-female ratio of case count (1.40:1) was similar to that in previous studies in Korea (23) and Japan (28) but slightly lower than that in Taiwan (29), Australia (28, 30), the United Kingdom (28), and Sweden (28). The intrinsic characteristics of KD, such as different expressions of interleukin-1 beta (IL-1β) (31) and genetic factors (32) by sex, have been suspected of sex-specific susceptibility of KD. In addition, studies in Japan suggest that various etiological candidates, including infectious triggers, sex hormones, and genetic and environmental factors, could explain seasonal variation by sex (33) and a decrease in the male-to-female ratio as age increases (34), which are concordant with our results. However, previous studies could not fully explain the sex-specific features of KD when a spatiotemporal perspective was involved. For example, the proportion of new and sporadic hot spots among total hot spots was higher in females than in males. The reason for the difference could derive from the temporal variation by sex, for which multiple factors are suspected. Additionally, the male-to-female ratio of KD decreased from the 2014–2015 period in Jeju-do, i.e., the island located at the southern end of the Korean Peninsula. Since the 2014–2015 period, KD cases on the island have gradually shifted to female dominance, and new hot spots in females have been identified, while the overall incidence has increased in both sexes. Considering that Jeju-do has a distinct subtropical climate regime unlike the Korean Peninsula and is more susceptible to climate change than other regions in South Korea (35), changes in environmental factors, such as temperature (36), precipitation, and wind, could be possible factors for the change in the sex ratio of Jeju-do. Therefore, the sex difference in KD implicates the possibility of multifactorial etiology.

Therefore, more careful analysis to explore multiple factors that result in sex differences along the spatiotemporal axis is required, as criticized. Low et al. explored the association between KD and numerous environmental and infectious factors in Canada by considering spatiotemporal effects; however, a lower number of KD cases precluded age and sex stratification (5). A longitudinal study in Japan that presents an insignificant decrease in KD admission during the COVID-19 pandemic quarantine indicates the possibility of other factors besides infectious triggers, while the lack of data also precluded the stratification of study subjects (37). Adding a spatial perspective and proper stratification to temporal associations that have been explored in previous studies between KD and infectious diseases [e.g., varicella and respiratory infections caused by rhinoviruses (9, 38) and respiratory syncytial virus (9), human bocaviruses (10) and enteroviruses (10, 38), pertussis (39, 40), and human coronaviruses (38, 41)] or environmental factors [e.g., air pollutants (13, 42) and temperature (42)] might help understand the etiology of KD.

Our findings support the etiologic hypothesis with multiple factors, including infectious, genetic, and environmental factors, by standing along with other observational and experimental studies, although our study had the limitation of being an ecological and descriptive study that could not imply causal inference. Furthermore, as we used the municipal level as the analysis unit, individual data that could consider individual demographic or clinical features is encouraged to be used for future analysis. However, the municipal level was the smallest unit available in the database, and we attempted to overcome this limitation by stratifying with sex and using a diverse spatial weight matrix. By using a diverse spatial weight matrix, it was possible to detect spatiotemporal clusters under different assumptions of the neighborhood. Furthermore, several studies used the municipal level to explore disease patterns (14, 43). Despite these limitations, by conducting spatiotemporal analysis with sex stratification, our study adds to the growing body of evidence for the etiologic hypothesis of multiple factors, including infectious triggers, and recommends future analysis directions.

## Data Availability

The data analyzed in this study are subject to the following licenses/restrictions: The data that support the findings of this study are available from the Korean National Health Insurance Service, but restrictions apply to the availability of these data, which were used under license for the current study, and so are not publicly available. Data are however available from the authors upon reasonable request and with permission of the Korean National Health Insurance Service. Requests to access these datasets should be directed to the corresponding author.
