# Supplementary material for: Spatiotemporal clusters of Kawasaki disease in South Korea from 2008 to 2017: A municipal-level ecological study
Source: Front Pediatr. 2023 Jan 24;10:1054985. doi: 10.3389/fped.2022.1054985 (PMC9904408; doi:10.3389/fped.2022.1054985)
Supplement: Supplementary file 1 [file Datasheet1.pdf]

frontiers

Supplementary Material

Supplementary Table 1. Patterns of emerging hot spot analysis based on [ESRI. How Emerging Hot Spot Analysis works. Available online: https://pro.arcgis.com/en/pro-app/2.9/tool-reference/space-time-pattern-mining/learnmoreemerging.htm (accessed on 16 March 2022)]

Pattern	Pattern name	Definition
	No Pattern Detected	Not applicable to any patterns defined below
	New Hot Spot	A location has a statistically significant hot spot in the final time step and has never been a statistically significant hot spot before the final time step
	Consecutive Hot Spot	A location has a single uninterrupted run of at least two statistically significant hot spot bins in the final time-step intervals and has never been a statistically significant hot spot before the final hot spot run, and less than 90% of all bins are statistically significant hot spots
	Intensifying Hot Spot	A location has been a statistically significant hot spot for 90% of the time-step intervals, including the final time step, and the intensity of clustering in each time step is statistically significantly increasing overall
	Persistent Hot Spot	A location has been a statistically significant hot spot for 90% of the time-step intervals without an identifiable increasing or decreasing trend in the intensity of clustering over time
	Diminishing Hot Spot	A location has a statistically significant hot spot for 90% of the time-step intervals, including the final time step, but the intensity of clustering in each step is statistically significantly decreasing overall
	Sporadic Hot Spot	A location with an on-again, off-again hot spot, with less than 90% of the time-step intervals being statistically significant hot spots and never having the time-step intervals of statistically significant cold spots
	Oscillating Hot Spot	A location is a statistically significant hot spot for the final time-step interval, but has a statistically significant cold spot during a prior time step and has less than 90% of the time-step intervals being statistically significant hot spots
	Historical Hot Spot	A location is not a hot spot in the most recent time period, but at least 90% of the time-step intervals have been statistically significant hot spots

	New Cold Spot	A location has a statistically significant cold spot in the final time step and has never been a statistically significant cold spot before the final time step
	Consecutive Cold Spot	A location has a single uninterrupted run of at least two statistically significant cold spot bins in the final time-step intervals and has never been a statistically significant cold spot before the final cold spot run, and less than 90% of all bins are statistically significant cold spots
	Intensifying Cold Spot	A location has been a statistically significant cold spot for 90% of the time-step intervals, including the final time step, and the intensity of clustering in each time step is statistically significantly increasing overall
	Persistent Cold Spot	A location has been a statistically significant cold spot for 90% of the time-step intervals without an identifiable increasing or decreasing trend in the intensity of clustering over time
	Diminishing Cold Spot	A location has a statistically significant cold spot for 90% of the time-step intervals, including the final time step, but the intensity of clustering in each step is statistically significantly decreasing overall
	Sporadic Cold Spot	A location with an on-again, off-again cold spot, with less than 90% of the time-step intervals being statistically significant cold spots, and never having the time-step intervals of statistically significant hot spots
	Oscillating Cold Spot	A location is a statistically significant cold spot for the final time-step interval, but has a statistically significant hot spot during a prior time step, with less than 90% of the time-step intervals being statistically significant cold spots
	Historical Cold Spot	A location is not a cold spot in the most recent time period, but at least 90% of the time-step intervals have been statistically significant cold spots

Supplementary Table 2. The name and the number of municipalities identified as hot or cold spots using emerging hot spot analysis for the Kawasaki disease incidence per 100,000 population by each time period

	Total sex	Male	Female
New hot spot	Changnyeong-gun, Uljin-gun, Yeongyang-gun (3)	Gangneung-si, Gyeongju-si, Nam-gu (Pohang-si), Uljin-gun, Yeongyang-gun (5)	Buk-gu (Pohang-si), Changnyeong-gun, Cheongsong-gun, Gimpo-si, Goseong-gun, Inje-gun, Jeju-si, Seogwipo-si, Uiryeong-gun (9)
Consecutive hot spot	Bucheon-si, Dongdaemun-gu, Donghae-si, Dongjak-gu, Gangdong-gu, Gangnam-gu, Gangneung-si, Geumcheon-gu, Gimpo-si, Goseong-gun, Guri-si, Guro-gu, Gwacheon-si, Gwanak-gu, Gwangjin-gu, Gwangmyeong-si, Gyeongju-si, Gyeyang-gu, Inje-gun, Jung-gu, Jungnang-gu, Jungwon-gu (Seongnam-si), Nam-gu (Pohang-si), Nowon-gu, Pyeongchang-gun, Seocho-gu, Seo-gu, Seongbuk-gu, Seongdong-gu, Sokcho-si, Songpa-gu, Sujeong-gu (Seongnam-si), Yangyang-gun, Yeongdeungpo-gu, Yongsan-gu (35)	Dongdaemun-gu, Dongjak-gu, Gangdong-gu, Gangnam-gu, Gangseo-gu, Geumcheon-gu, Gwacheon-si, Gwanak-gu, Gwangjin-gu, Gyeyang-gu, Ilsanseo-gu (Goyang-si), Jongno-gu, Jung-gu, Jungnang-gu, Mapo-gu, Seocho-gu, Seodaemun-gu, Seongbuk-gu, Seongdong-gu, Sokcho-si, Songpa-gu, Yangcheon-gu, Yangyang-gun, Yeongdeungpo-gu, Yongsan-gu (25)	Bucheon-si, Dongdaemun-gu, Donghae-si, Dongjak-gu, Gangnam-gu, Gangneung-si, Geumcheon-gu, Guri-si, Gwanak-gu, Gwangmyeong-si, Gyeongju-si, Gyeyang-gu, Jongno-gu, Jung-gu, Jungnang-gu, Mapo-gu, Nam-gu (Pohang-si), Seocho-gu, Seodaemun-gu, Seongbuk-gu, Seongdong-gu, Sokcho-si, Sujeong-gu (Seongnam-si), Yanggu-gun, Yangyang-gun, Yeongdeungpo-gu, Yongsan-gu (27)
Sporadic hot spot	Deogyang-gu (Goyang-si), Dobong-gu, Eunpyeong-gu, Gangbuk-gu, Gangseo-gu, Ilsandong-gu (Goyang-si), Ilsanseo-gu (Goyang-si), Jongno-gu, Mapo-gu, Seodaemun-gu, Hongseong-gun, Yangcheon-gu, Yesan-gun (13)	Hongseong-gun, Yesan-gun, Guro-gu, Ilsandong-gu (Goyang-si), Deogyang-gu (Goyang-si), Eunpyeong-gu, Gangbuk-gu (7)	Deogyang-gu (Goyang-si), Eunpyeong-gu, Gangseo-gu, Guro-gu, Ilsandong-gu (Goyang-si), Ilsanseo-gu (Goyang-si), Nowon-gu, Pyeongchang-gun, Yangcheon-gu (9)

New cold spot	Gurye-gun, Namwon-si, Sinan-gun (3)	Gurye-gun, Namwon-si (2)	Sinan-gun (1)
Sporadic cold spot	Goheung-gun, Gokseong-gun (2)	Gokseong-gun (1)	Ongjin-gun (1)

The value in the bracket indicates the number of municipalities detected as hot or cold spots

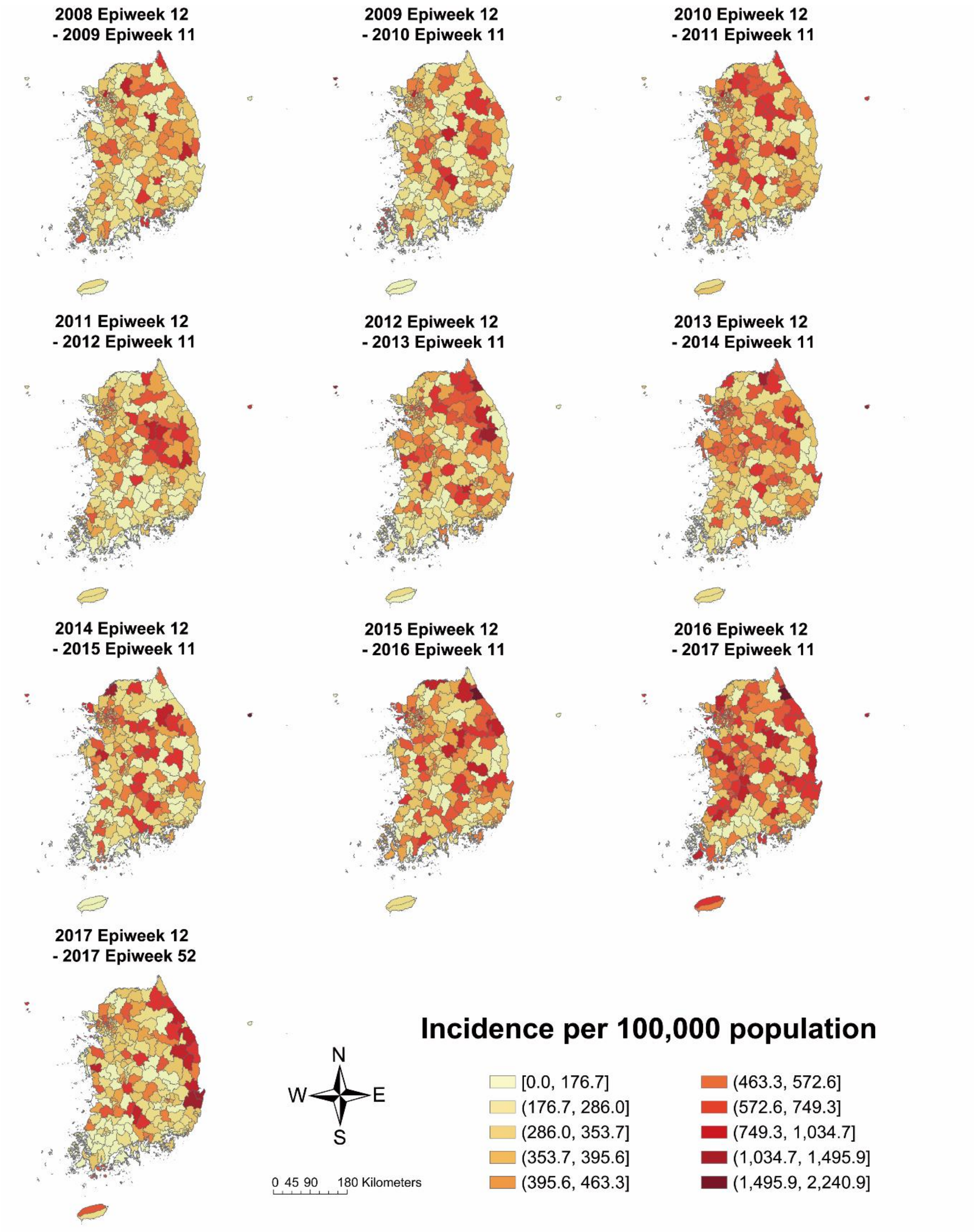

Supplementary Figure 1. Kawasaki disease incidence per 100,000 population on male subjects by each time period. *The 2017 Epiweek 12 to 2017 Epiweek 52 period includes a partial year, i.e., 41 weeks.*

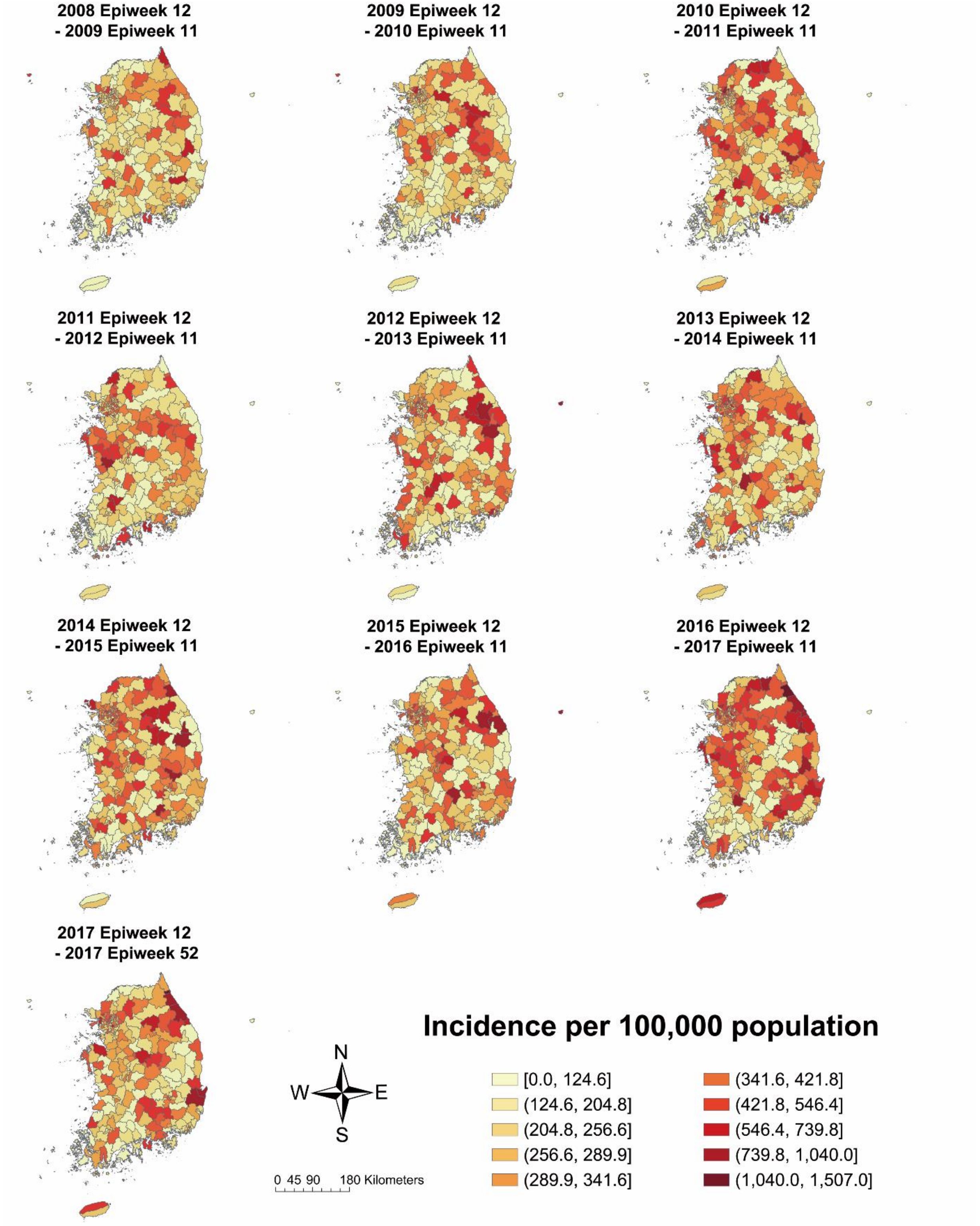

Supplementary Figure 2. Kawasaki disease incidence per 100,000 population on female subjects by each time period. *The 2017 Epiweek 12 to 2017 Epiweek 52 period includes a partial year, i.e., 41 weeks.*

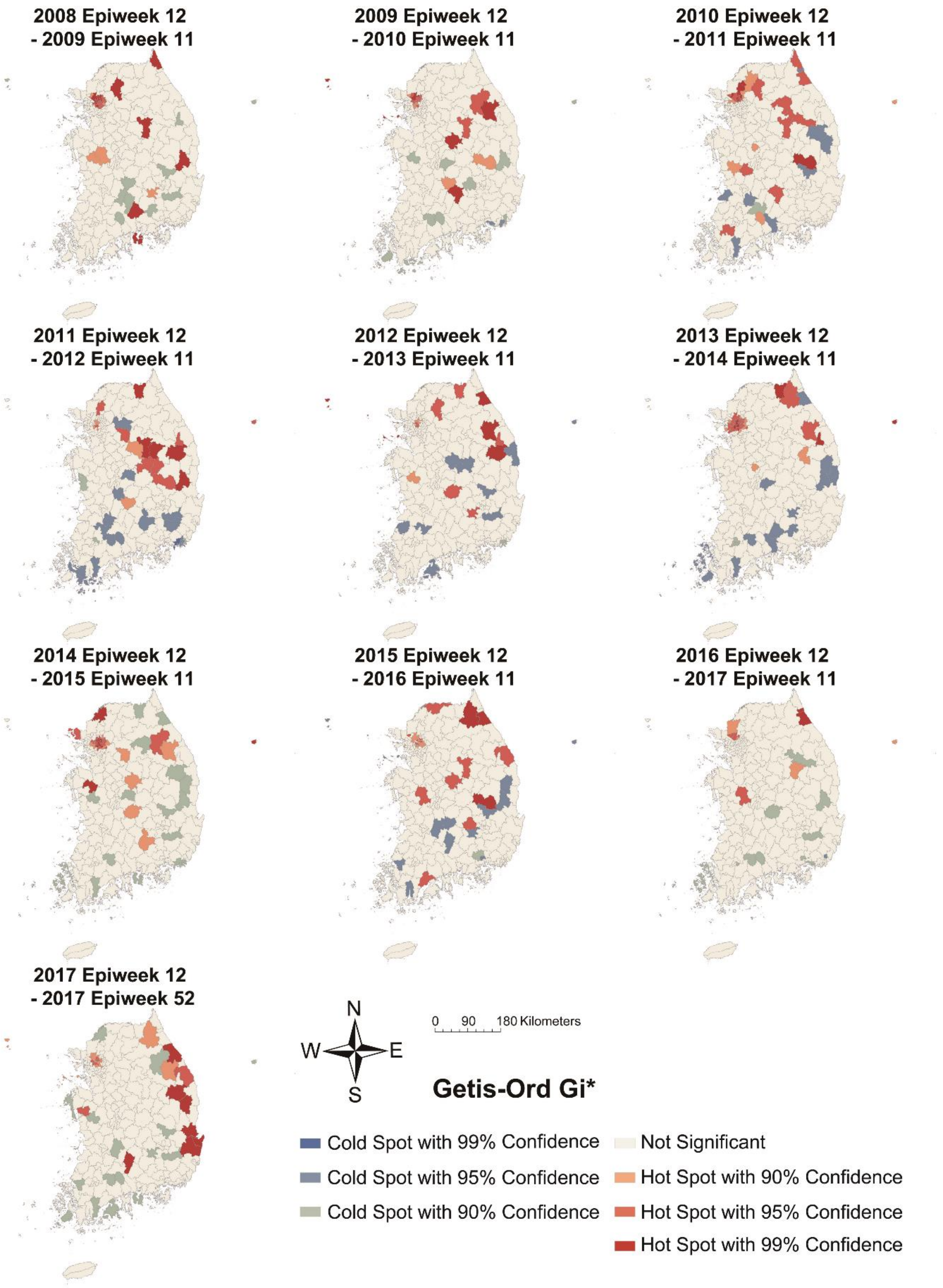

Supplementary Figure 3. Clusters identified by Getis-Ord Gi* analysis for Kawasaki disease incidence per 100,000 population on males by each year-time period. *Colors represent hot spots and cold spots of spatial clustering with 90, 95, and 99% confidence intervals. The 2017 Epiweek 12 to 2017 Epiweek 52 period includes a partial year, i.e., 41 weeks.*

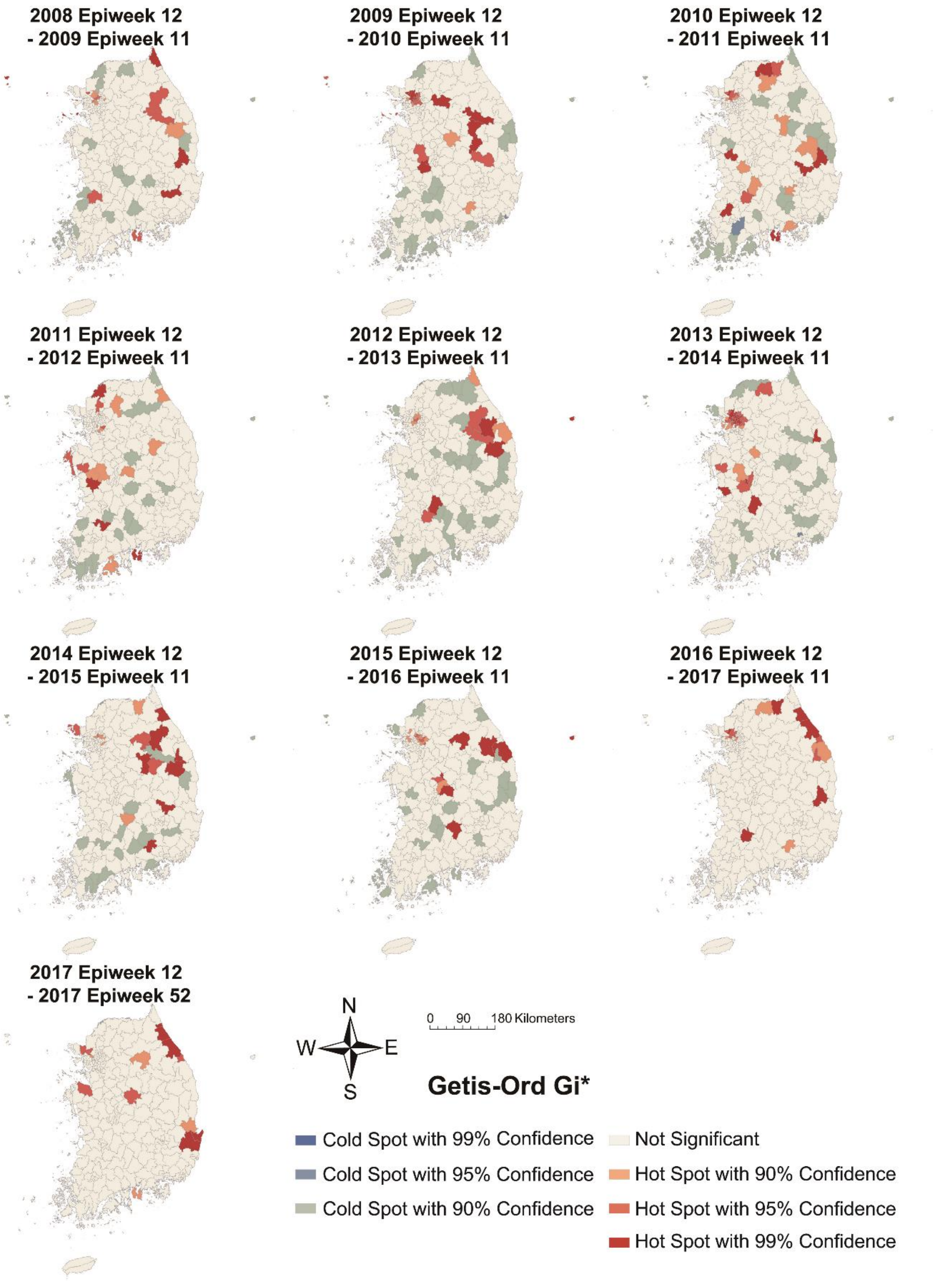

Supplementary Figure 4. Clusters identified by Getis-Ord Gi* analysis for Kawasaki disease incidence per 100,000 population on females by each year-time period. *Colors represent hot spots and cold spots of spatial clustering with 90, 95, and 99% confidence intervals. The 2017 Epiweek 12 to 2017 Epiweek 52 period includes a partial year, i.e., 41 weeks.*

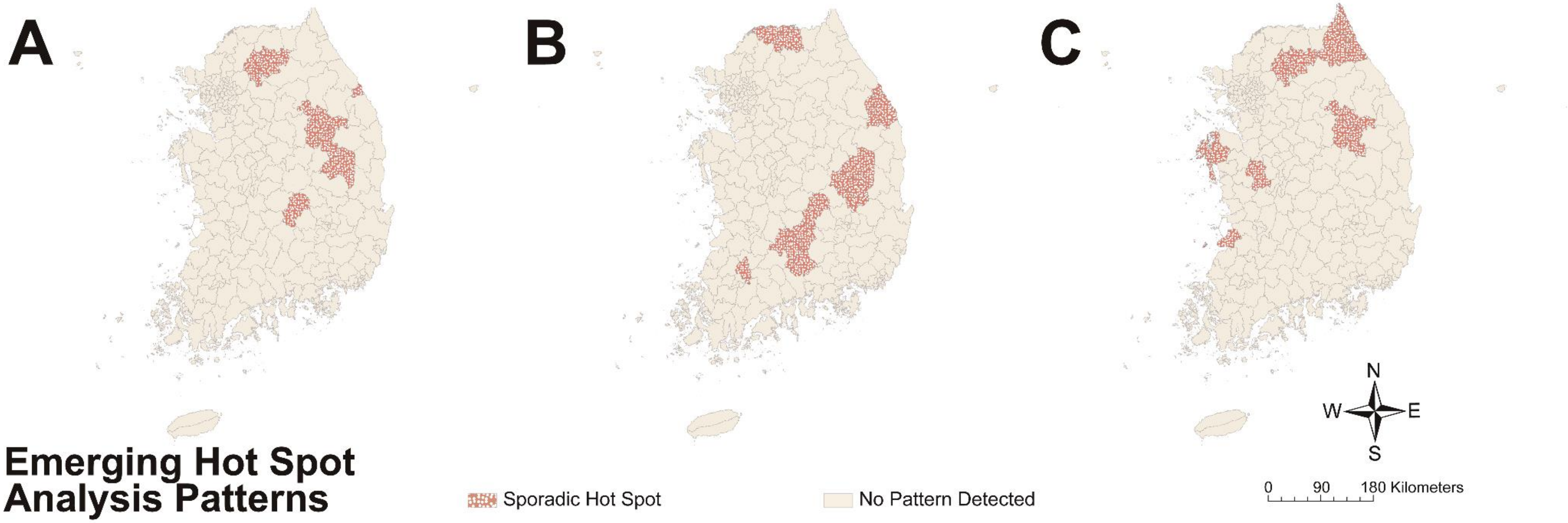

Supplementary Figure 5. Hot or cold spot area detected by emerging hot spot analysis for Kawasaki disease incidence per 100,000 population by week from 2008 Epiweek 12 to 2017 Epiweek 52 on **(A)** total study subject, **(B)** male, and **(C)** female. *Colors represent the patterns of hot or cold spots (refer to Supplementary Table 1).*

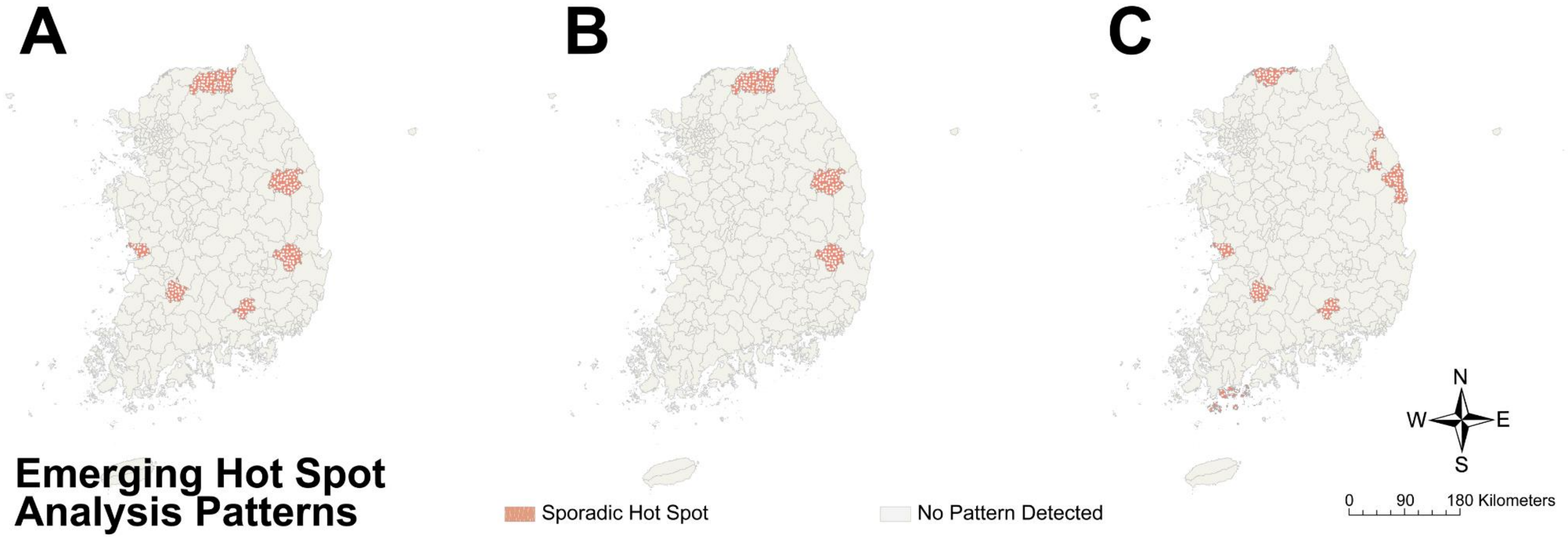

Supplementary Figure 6. Hot or cold spot area detected by emerging hot spot analysis for Kawasaki disease incidence per 100,000 population by week from 2008 Epiweek 12 to 2017 Epiweek 11 on **(A)** total study subject, **(B)** male, and **(C)** female. *Colors represent the patterns of hot or cold spots (refer to Supplementary Table 1).*

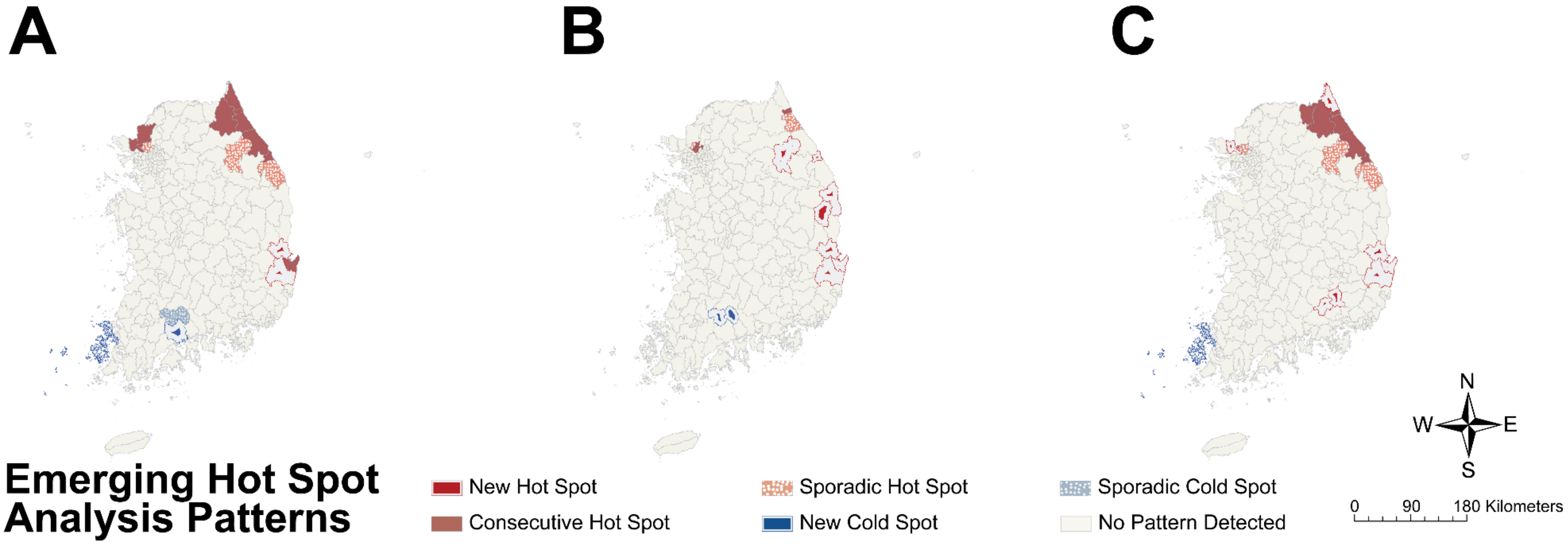

Supplementary Figure 7. Hot or cold spot area detected by emerging hot spot analysis for Kawasaki disease incidence per 100,000 population by each time period using row standardized spatial weight matrix with setting K nearest neighbor as two on **(A)** total study subject, **(B)** male, and **(C)** female. *Colors represent the patterns of hot or cold spots (refer to Supplementary Table 1).*
